# Ceftriaxone-induced up-regulation of cortical and striatal GLT1 in the R6/2 model of Huntington's disease

**DOI:** 10.1186/1423-0127-17-62

**Published:** 2010-07-27

**Authors:** Youssef Sari, Anne L Prieto, Scott J Barton, Benjamin R Miller, George V Rebec

**Affiliations:** 1 Program in Neuroscience, Indiana University, 1101 East 10th Street, Bloomington, IN, USA; 2 Department of Psychological and Brain Sciences, Indiana University, 1101 East 10th Street, Bloomington, IN, USA; 3 University of Toledo, College of Pharmacy, Department of Pharmacology, Health Science Campus, 3000 Arlington Avenue, Toledo, OH 43606, USA; 4 University of Texas Southwestern Medical School, Department of Physiology. 5323 Harry Hines Boulevard, Dallas, TX 75390, USA

## Abstract

**Background:**

Huntington's disease (HD) is an inherited neurodegenerative disorder characterized by cortico-striatal dysfunction and loss of glutamate uptake. At 7 weeks of age, R6/2 mice, which model an aggressive form of juvenile HD, show a glutamate-uptake deficit in striatum that can be reversed by treatment with ceftriaxone, a β-lactam antibiotic that increases GLT1 expression. Only at advanced ages (> 11 weeks), however, do R6/2 mice show an actual loss of striatal GLT1. Here, we tested whether ceftriaxone can reverse the decline in GLT1 expression that occurs in older R6/2s.

**Results:**

Western blots were used to assess GLT1 expression in both striatum and cerebral cortex in R6/2 and corresponding wild-type (WT) mice at 9 and 13 weeks of age. Mice were euthanized for immunoblotting 24 hr after five consecutive days of once daily injections (ip) of ceftriaxone (200 mg/kg) or saline vehicle. Despite a significant GLT1 reduction in saline-treated R6/2 mice relative to WT at 13, but not 9, weeks of age, ceftriaxone treatment increased cortical and striatal GLT1 expression relative to saline in all tested mice.

**Conclusions:**

The ability of ceftriaxone to up-regulate GLT1 in R6/2 mice at an age when GLT1 expression is significantly reduced suggests that the mechanism for increasing GLT1 expression is still functional. Thus, ceftriaxone could be effective in modulating glutamate transmission even in late-stage HD.

## Background

Ample evidence indicates that the neuropathology associated with Huntington's disease (HD), an autosomal dominant condition characterized by behavioral, cognitive, and physical deterioration, involves the dysregulation of glutamate, an excitatory amino acid [1-4]. In fact, a decline in glutamate removal has been observed in the brains of transgenic mouse models of HD [5-7] as well as HD patients post-mortem [8]. Loss of glutamate uptake leads to accumulation of extracellular glutamate, making neurons vulnerable to excitotoxicity. Interestingly, GLT1, a protein expressed primarily on glial cells and responsible for the removal of most extracellular glutamate [9,10], appears to be dysfunctional in HD mouse models [5,6,11]. We recently reported that the deficit in glutamate uptake in the commonly used R6/2 model at 8 weeks of age can be reversed following treatment with ceftriaxone [7], a beta-lactam antibiotic that elevates the level of GLT1 without altering the expression of other glutamate transporters [12]. By up-regulating GLT1, ceftriaxone appears to overcome a functional GLT1 deficit since the level of protein does not decline until R6/2 mice exceed 11 weeks of age [5,6,11]. Here, we determined if ceftriaxone could increase GLT1 expression even in R6/2 mice that have a deficit in GLT1 production. We focused on cerebral cortex and striatum, two forebrain regions that show the greatest HD neuropathology [13,14]. Our results suggest that the cellular machinery by which ceftriaxone increases cortical and striatal GLT1 expression is still intact even in late-stage HD.

## Methods

### Animals

Male transgenic R6/2 mice (B6CBA-TgN[HDexon1]62Gpb) and wild-type (WT) controls were obtained from The Jackson Laboratories (Bar Harbor, ME) at 6 weeks of age. All mice were housed individually in the departmental animal colony under standard conditions (12 hr light/dark cycle with lights on at 07:00 AM) with access to food and water *ad libitum*. Both the housing and experimental use of animals followed the National Institutes of Health guidelines and were approved by the Institutional Animal Care and Use Committee at Indiana University Bloomington.

### Genotype and CAG repeat length

We used PCR for genotyping and characterizing the CAG repeat length as previously reported [7]. Our R6/2 mice had 121 ± 1.8 (mean ± SEM) CAG repeats, which is within the range for developing the HD behavioral phenotype [15].

### Treatment protocol

R6/2 and WT mice at either 8 or 12 weeks of age were weighed and injected ip with 200 mg/kg ceftriaxone (Sigma, St. Louis, MO) or an equal volume of saline once daily for 5 consecutive days. Twenty-four hours after the last injection, when the mice had reached 9 or 13 weeks of age, the animals were decapitated. Their brains were removed, and cerebral cortex and striatum from both hemispheres were dissected and frozen for immunoblotting.

### Western blot

Western blots for GLT1 detection were performed as previously described [7,16]. In brief, extracted proteins were separated in 4-20% glycine gel (Invitrogen). The membranes were blocked in 3% milk in TBST (50 mM Tris HCl; 150 mM NaCl, pH7.4; 0.1% Tween20) for 30 min at room temperature. The membranes were then incubated with guinea pig anti-GLT1 antibody (Millipore Bioscience Research Reagents) at 1:5,000 dilution in blocking buffer at 4˚C. After washing and blocking, the membranes were incubated with horseradish peroxidase (HRP)-labeled anti-guinea pig secondary antibody (1:10,000 dilution) in the blocking buffer. Protein loading was normalized using β-tubulin immunoblotting as a loading control. Chemiluminescent detection of HRP (SuperSignal West Pico; Pierce) was followed by exposure of the membranes to a Kodak BioMax MR film (Thermo Fisher Scientific). The films were developed on an SRX-101A machine. Digitized images of immunoreactive proteins were quantified using an MCID system. The data are reported as percentage ratios of GLT1/β-tubulin.

### Statistical analysis

Data were analyzed by means of a two-way analysis of variance (ANOVA) and Bonferroni *post hoc *tests. All statistical tests required a level of significance of at least *P *< 0.05.

## Results

### Body weights

Table 1 shows the mean body weight of all groups on the last day of treatment. No significant differences were found between genotype (WT and R6/2) or treatment group (ceftriaxone and saline) at 9 weeks of age. Regardless of treatment, however, there was a significant reduction in body weight in R6/2 relative to WT mice (*P *< 0.001) at 13 weeks of age, which supports previous evidence that at this age R6/2 mice are strongly symptomatic [17].

**Table 1:** Body weight

Age	WTs	R6/2s	WTc	R6/2c
**9-week**	27.53 ± 1.18 (N = 4)	26.52 ± 1.02 (N = 4)	27.27 ± 1.02 (N = 4)	28.06 ± 1.18 (N = 4)
**13-week**	34.3 ± 2.75 (N = 5)	*26.78 ± 1.69 (N = 5)	33.00 ± 0.98 (N = 5)	*24.52 ± 2.33 (N = 5)

Data are presented as mean body weight (g) ± SEM. * *P *< 0.001, HD compared to their respective WT. Abbreviations: WTs and R6/2s indicate saline treatment, and WTc and R6/2c indicate ceftriaxone treatment. N refers to number of animals per group.

### Effects of ceftriaxone treatment in cortical and striatal GLT1 expression

**Figure 1:**
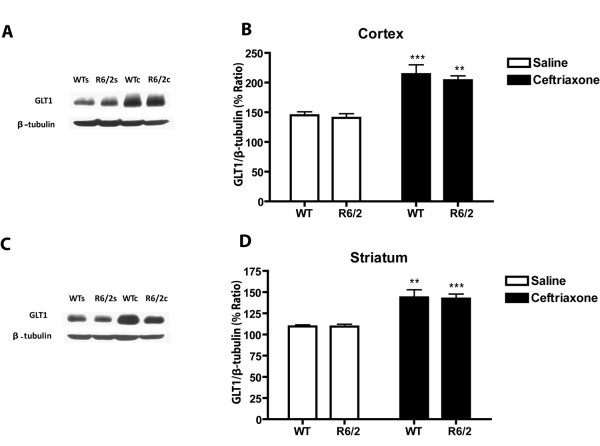
**Effects of ceftriaxone on GLT1 expression in cerebral cortex and striatum at 9 weeks of age**. Immunoblots **(A, C) **and quantitative analysis **(B, D) **of the percentage ratio of GLT1/β-tubulin in cerebral cortex and striatum, respectively (***P < 0.001 and **P < 0.01 relative to corresponding saline group). Error bars indicate SEM. (N = 4 for each group).

**Figure 2:**
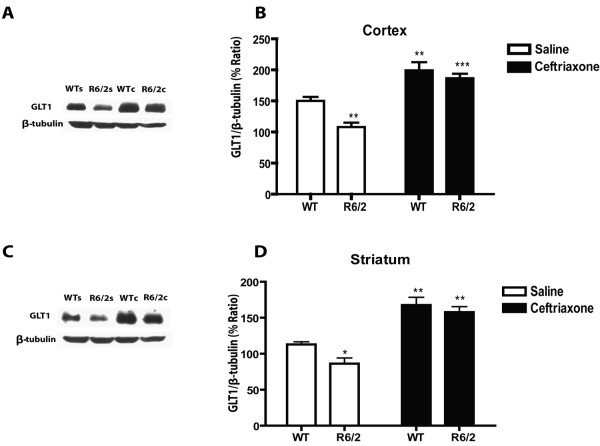
**Effects of ceftriaxone on GLT1 expression in cerebral cortex and striatum at 13 weeks of age**. Immunoblots **(A, C) **and quantitative analysis **(B, D) **of the percentage ratio of GLT1/β-tubulin in cerebral cortex and striatum, respectively (**P < 0.01 and ***P < 0.001 relative to corresponding saline group). Note the reduction in the percentage ratio of GLT1/β-tubulin expression in cerebral cortex (**P < 0.01) and striatum (*P < 0.05) of saline-treated R6/2 mice relative to saline-treated WT mice, and the elimination of this effect after ceftriaxone. Error bars indicate SEM. (N = 5 for each group).

Although saline-treated R6/2s showed no loss of either cortical or striatal GLT1 relative to WT at 9 weeks of age (Figure 1), there was a marked reduction in both brain regions in similarly treated 13-week-old R6/2s (Figure 2). Quantitative analysis of this age group revealed significant genotypic differences in GLT1 expression in both cerebral cortex (*P *< 0.01) and striatum (*P *< 0.03). Despite the loss of GLT1 in older R6/2s, these animals showed the same response to ceftriaxone as the younger R6/2s and both WT age groups. Thus, WT and R6/2 mice at either 9 (Figure 1) or 13 weeks of age (Figure 2) responded to ceftriaxone with an increase in cortical and striatal GLT1 expression relative to saline. Quantitative analysis revealed a significant effect of ceftriaxone in both brain regions at 9 and 13 weeks of age (*P *< 0.0001 in each case).

## Discussion

Our results not only confirm the ability of ceftriaxone to elevate GLT1 expression in cortex and striatum of R6/2 mice, but show that this effect still occurs even after GLT1 levels begin to decline when these mice are 13 weeks of age and severely symptomatic. Thus, it appears that the cellular machinery underlying the ceftriaxone-induced increase in GLT1 expression is operative in late-stage HD.

Although the mechanism by which ceftriaxone increases GLT1 expression is not clear, there is support for activation of nuclear factor-kappa B (NF-kB), a transcription factor that plays a role in regulating immune responses and cell survival [18]. Translocation of the NF-kB complex to the cell nucleus appears to be critical for the action of ceftriaxone [19], and our results suggest that this mechanism is intact in both cortex and striatum of R6/2 mice regardless of age. Even before the decline in GLT1 expression, moreover, 8-week-old R6/2 mice have a deficit in glutamate uptake, which is reversed by ceftriaxone treatment [7]. Although there is no GLT1 protein deficit at this age, mRNA levels are in decline [6] and glutamate uptake is reduced [7], suggesting a loss of transporter function well in advance of protein down-regulation. Thus, ceftriaxone is capable of overcoming a deficit in GLT1 function. It is interesting in this regard that palmitoylation, a process by which proteins are inserted into cellular membranes [20], is reduced in HD mice, including palmitoylation of GLT1 [21]. Whether ceftriaxone increases GLT1 palmitoylation is the focus of ongoing research.

It is unlikely that other glutamate transporters can account for a ceftriaxone-induced increase in glutamate uptake since ceftriaxone acts selectively on GLT1 [12]. It also is unlikely that loss of other glutamate transporters can account for the decline in uptake since neither mRNA nor protein levels are altered for GLAST and EAAC1 in HD models even at ages when the behavioral phenotype is severe [6]. Post-mortem analysis of HD patients, moreover, shows a selective decline in GLT1 mRNA expression [22] as well as a loss of glutamate uptake [8]. Nevertheless, we cannot rule out the possibility that ceftriaxone has other actions that may indirectly impact glutamate transmission, including a change in dopamine or GABA dynamics. Although an antibiotic action of ceftriaxone is unlikely in that none of our animals showed signs of sepsis, it would be useful in follow-up studies to determine if non-antibiotics that also up-regulate GLT1, such as GPI-1046 [23], mimic the effects of ceftriaxone in R6/2 mice.

Increasing GLT1 expression may become an effective HD treatment strategy in that the up-regulation of GLT1 induced by ceftriaxone significantly improves the behavioral phenotype in 8-week-old R6/2 mice [7]. It is unlikely that starting ceftriaxone treatment in 13-week-old R6/2s will result in behavioral improvement given the stage of disease progression in these animals, and in fact, we found that ceftriaxone failed to reverse the decline in body weight, which is evident in R6/2s at this age. But our results suggest that the increase in GLT1 expression that occurs when ceftriaxone treatment is begun earlier will continue to occur even in late-stage HD. Thus, GLT1 expression is likely to be an effective therapeutic target over a relatively long time course.

Glutamate dysregulation, including a possible decline in GLT1 activity, may play a role in several neurodegenerative diseases [5,24]. In fact, a phase III clinical trial of ceftriaxone for treatment of amyotrophic lateral sclerosis (ALS) is already underway (for review see [25]). The dose required to increase GLT1 in mice produces comparable levels of ceftriaxone in the central nervous system of patients undergoing treatment for meningitis (0.3-6 μmol/L) [26], indicating that our treatment protocol is within normal limits for this drug. Nevertheless, it is interesting that ceftriaxone increased cortical and striatal GLT1 expression in both R6/2 and WT mice. WT mice, however, show no discernable behavioral consequences [7], suggesting that mechanisms are in place to compensate for increased glutamate removal. Whether HD mice lack these mechanisms or simply benefit from an increased rate of glutamate uptake remains to be determined. It appears that within limits increased GLT1 expression is not a problem, but decreased expression, which occurs in HD, is.

## Conclusions

Ceftriaxone treatment enhances GLT1 expression in cerebral cortex and striatum of R6/2 mice at 13 weeks of age when endogenous GLT1 levels decline. These data suggest that the mechanism for increasing GLT1 expression is still functional even in late stage HD.

## Competing interests

The authors declare that they have no competing interests.

## Authors' contributions

YS participated in study design and conceptualization, collected and analyzed data, helped with data interpretation, and drafted the manuscript. ALP helped with data collection, analysis, and interpretation. SJB performed statistical analyses and genotyping, and helped with data interpretation. BRM participated in study design, and helped with data collection and analysis. GVR conceptualized and designed the study, and revised the manuscript for intellectual content. All authors read and approved the final manuscript.
